# Functional Assessment of Cardiac Responses of Adult Zebrafish (*Danio rerio*) to Acute and Chronic Temperature Change Using High-Resolution Echocardiography

**DOI:** 10.1371/journal.pone.0145163

**Published:** 2016-01-05

**Authors:** Ling Lee, Christine E. Genge, Michelle Cua, Xiaoye Sheng, Kaveh Rayani, Mirza F. Beg, Marinko V. Sarunic, Glen F. Tibbits

**Affiliations:** 1 Molecular Cardiac Physiology Group, Department of Biomedical Physiology and Kinesiology, Simon Fraser University, 8888 University Drive, Burnaby, BC V5A 1S6, Canada; 2 Child and Family Research Institute, Department of Cardiovascular Science, 950 West 28th Ave, Vancouver, BC V5Z 4H4, Canada; 3 School of Engineering Science, Simon Fraser University, 8888 University Drive, Burnaby, BC V5A 1S6, Canada; Mayo Clinic, UNITED STATES

## Abstract

The zebrafish (*Danio rerio*) is an important organism as a model for understanding vertebrate cardiovascular development. However, little is known about adult ZF cardiac function and how contractile function changes to cope with fluctuations in ambient temperature. The goals of this study were to: 1) determine if high resolution echocardiography (HRE) in the presence of reduced cardiodepressant anesthetics could be used to accurately investigate the structural and functional properties of the ZF heart and 2) if the effect of ambient temperature changes both acutely and chronically could be determined non-invasively using HRE *in vivo*. Heart rate (HR) appears to be the critical factor in modifying cardiac output (CO) with ambient temperature fluctuation as it increases from 78 ± 5.9 bpm at 18°C to 162 ± 9.7 bpm at 28°C regardless of acclimation state (cold acclimated CA– 18°C; warm acclimated WA– 28°C). Stroke volume (SV) is highest when the ambient temperature matches the acclimation temperature, though this difference did not constitute a significant effect (CA 1.17 ± 0.15 μL at 18°C vs 1.06 ± 0.14 μl at 28°C; WA 1.10 ± 0.13 μL at 18°C vs 1.12 ± 0.12 μl at 28°C). The isovolumetric contraction time (IVCT) was significantly shorter in CA fish at 18°C. The CA group showed improved systolic function at 18°C in comparison to the WA group with significant increases in both ejection fraction and fractional shortening and decreases in IVCT. The decreased early peak (E) velocity and early peak velocity / atrial peak velocity (E/A) ratio in the CA group are likely associated with increased reliance on atrial contraction for ventricular filling.

## Introduction

Ectothermic fish must cope with environmental temperature changes on a chronic (seasonal) and on an acute basis. Changes in water temperature can have drastic effects on both resting and maximum cardiac performance [1,2]. Only certain species of fish have the ability to tolerate significant acute temperature changes [3], typically eurythermal species with an evolutionary history of exposure (either on a daily basis and or via water column movements). A limiting factor in the ability to cope with these acute changes is adequate cardiac contractility, so species must possess sufficient myocardial plasticity [4]. Fish typically exhibit changes in heart rate with acute temperature fluctuation [2,5] but the extent to which this varies cardiac output depends on the species. Most changes that occur with acute temperature fluctuation are readily reversible. Acclimation, or remodeling to cope with longer term changes in temperature, typically involves transcriptional regulation which results in morphological or biochemical changes in order to cope with the new environment. Since these changes take longer to accomplish, there is often a cost associated with them, such as the energetic cost of producing proteins or an expression profile that is no longer optimal for the current environment.

While temperature effects on metabolic processes have been well documented, the overall morphological and functional changes to the adult teleost heart have not been as comprehensively studied. The teleost heart is composed of a series of four chambers: venous sinus; atrium; ventricle and bulbus arteriosus. These chambers develop from a simple linear tube [6] and differ not only morphologically but also physiologically with different contractility characteristics. Compared to mammals, the relative volume of the fish atrium is closer to that of the ventricle and despite being less developed muscularly than the ventricle, the teleost atrium plays a much more important role in the active filling of the ventricle. In both mammals and fish a key regulator of cardiac performance is the end diastolic volume (EDV). EDV in fish is greatly determined by active atrial contraction rather than by central venous pressure as seen in mammals [7]. Thus the modulation of atrial contractility as well as Frank-Starling mechanisms involving preload in the ventricle are particularly important for determining ventricle filling in teleosts. The reported degree to which the active role of the atrium has modulated cardiac output in the literature has varied depending on both the experimental set-up and the species [8,9,10,11]. Many of the morphological differences between chambers may have evolved in order to meet the high physiological demands of variation in metabolic rate and increased tolerance to a wide range of environmental conditions. Chamber-specific properties may lend themselves to teleost- specific functional responses to ambient temperature. Subjecting the heart to acute temperature stress allows one to look at the intrinsic ability of the fish heart to cope with rapid changes in temperature that do not allow for transcriptional level remodeling. Conversely, longer-term acclimation to temperature stress allows us to examine more extensive remodeling of the heart guided by changes in gene expression profiles.

Many previous studies have used a reductionist approach to determine the potential limitations of heart performance with respect to temperature; however, the variability in results due to experimental set-up makes it difficult to determine the *in vivo* effects on cardiac contraction. Conventionally, cardiac hemodynamics have been measured by invasive catheterization techniques in fish [10,12,13] but these involve manipulation of the *in situ* environment to mimic *in vivo* conditions leading to inconsistencies in the background. Stroke volume (SV) that is derived from cardiac output has been shown to underestimated from the measured SV typically by 15% in the teleost heart [10]. Doppler technology has been used to study intracardiac flows non-invasively, but its use in fish has been largely limited to surgically implanted probes in some cases requiring potential error-introducing calibration after removal from the animal [14], as well as being limited to fish larger than zebrafish.

The use of echocardiography with a high frequency (50–70 MHz) probe can allow for high resolution, real-time, noninvasive imaging to examine many parameters of cardiac structure and function. The zebrafish has been proposed by some to be a robust model of human cardiac electrophysiology due to the fact that its heart rate and action potential morphology are similar although some differences are apparent [15,16]. Zebrafish have a wide seasonal temperature range from 6°C to 38°C [17] and its high temperature tolerance make this real-time technique ideal for demonstrating how the cardiac function of eurythermal species cope with temperature fluctuation [18]. The use of conventional ultrasound equipment (7 and 8.5 MHz) in previous studies on zebrafish [12] had insufficient resolution leading to problems with visualization of specific chambers and specific valves, or conventional use of relatively high doses (100–200 ppm) of the fish anesthetic MS-222 [19] confounds measurements of HR and CO through its well-documented cardio-depressive effects (S1 Table) [12,19]. In this study we used high-resolution echocardiography using a more appropriate anesthetic to look at the structural and functional responses of the zebrafish heart to ambient temperature changes as a result of acute change and longer-term acclimation. We clearly demonstrate the ability of high-resolution echocardiography to accurately and non-invasively determine multiple parameters of cardiac structure and function *in vivo* in the adult zebrafish and firmly establish the utility of this technique in characterizing responses to environmental perturbations in similar-sized teleosts.

## Methods

### Animals and cold acclimation

All animal experiments were approved by the University of British Columbia Animal Care Committee (protocol number A12-0198) and adhered to the Canadian Council on Animal Care (CCAC) guidelines. Warm-acclimated (WA) adult zebrafish purchased from a local supplier were maintained at a 12-h:12-h light:dark photoperiod at 28°C in dechlorinated water in a flow-through tank and fed *ad libitum* (Nutrafin Max, Hagen Inc., Baie d’Urfé, QC, Canada). The zebrafish were, on average, ~3.8 cm in length at the time of echocardiography and were approximately 6 months of age. The cold-acclimated (CA) group of adult zebrafish was held at 28°C for two weeks in flow-through tanks, followed by a temperature decrease of 2.5°C/week until the end-point temperature of 18°C was reached. Fish were then held at this temperature for at least 4 weeks.

### Zebrafish holding conditions

For echocardiography, WA fish were held for 7 days in an aquarium in which the temperature was maintained at 28°C by means of an Eheim Jӓger aquarium thermostatic heater (Deizisau, Germany). CA fish were also held for 7 days in a chilled aquarium with the temperature maintained at 18°C. The aquarium water was double distilled and a GH Mineralized mineral supplement was added (Aquavitro, Madison GA) in a ratio of 1 ml / 8 L to maintain minimum calcium and magnesium concentrations of 13.5% and 1.2%, respectively. The pH was adjusted to 7.2 with NaOH.

### Anaesthetic preparation

MS-222 (Tricaine methane sulfonate) is used extensively as an anesthetic in fish studies, however at the concentrations typically used (100–200 ppm or mg/L), it acts as a profound cardiodepressant [20] and can seriously confound the results. In this study a combination of 45 ppm of pH-adjusted MS-222 (Sigma-Aldrich, Oakville, ON) and 45 ppm isoflurane (Sigma-Aldrich, Oakville, ON) was used for zebrafish to allow anaesthesia time to be extended with less consequence to cardiac function in comparison to MS-222 alone [21]. To create stock solutions, MS-222 was dissolved in distilled water to a final concentration of 2,000 ppm and buffered to pH 7.4 with NaOH and isoflurane was dissolved in 100% ethanol in a ratio of 1:9 to a final concentration of 100,000 ppm. Zebrafish were placed in a 45 ml custom-made water-jacketed glass chamber containing 30 ml of aquarium water and an initial concentration of 30 ppm of each drug. After 15 minutes, a second dose of MS-222 and isoflurane anesthetic mix (15 ppm) was added to yield final concentrations of 45 ppm of each drug. It was determined that the appropriate anesthetised condition for echocardiography was reached when the zebrafish were unresponsive to a slight tail pinch with forceps. Breathing was monitored by visual tracking of opercular movement to ensure fish health throughout the protocol.

### Echocardiography

**Fig 1:**
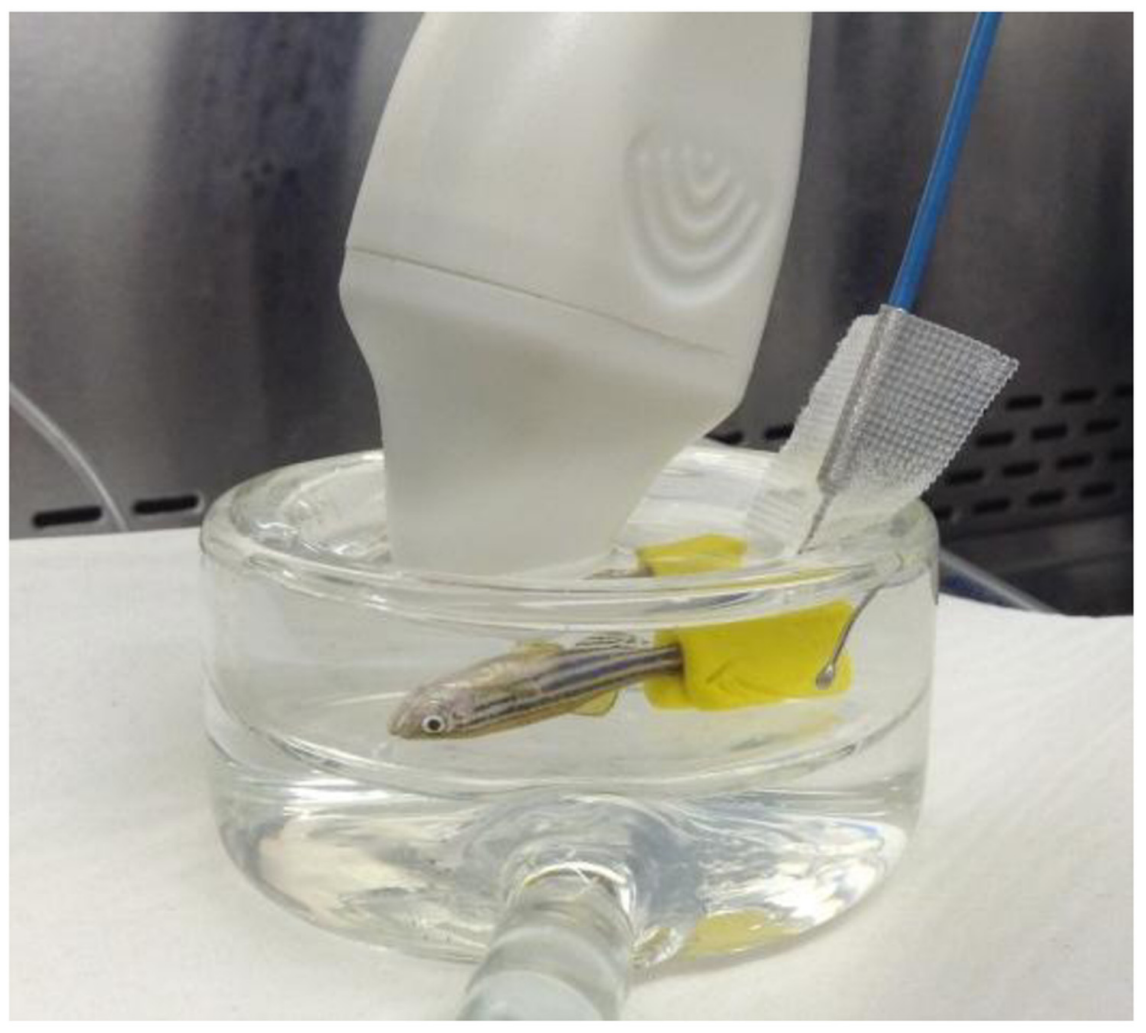
Longitudinal position of zebrafish. The ultrasound transducer beam (70 MHz) was positioned at about 85 degrees to ventral surface of the anesthetized zebrafish through all protocols. ZF were placed in a 45 ml custom-made water-jacketed glass chamber with the thermal probe (shown with the blue wire) inserted into the bath in proximity to the ZF.

Anesthetized adult zebrafish (n = 10 for each treatment group) were held by a small piece of modelling clay (Play-Doh™ Hasbro, Longueuil, QC) on the tail and placed ventral side up in the glass chamber. The water jacket was connected to a water bath to ensure precise temperature control. Aquarium water temperature was monitored by a temperature probe submerged in the bath chamber. Echocardiographs of the zebrafish were acquired by using a Vevo 2100 ultrasound system (VisualSonics®, Toronto, ON, Canada), with a 70 MHz ultrasound transducer fixed above the ventral side of the zebrafish and parallel to the longitudinal axis plane (Fig 1). The frame rate ranged from 20 to 120 (depending on image size) frames per second. The specifications of the linear array probe (Model MS700) used in this study included having a central frequency of 50 MHz, bandwidth from 30–70 MHz, a focal length of 5.0 mm and a maximum frame acquisition rate of 476 fps (single zone, 4.73 mm width, B-mode). The maximum field of view of 2D imaging was 9.7 x 12.0 mm with a spatial resolution of 75 μm (lateral) by 30 μm (axial). All calculations were done using the cardiac package of the VisualSonics software.

**Fig 2:**
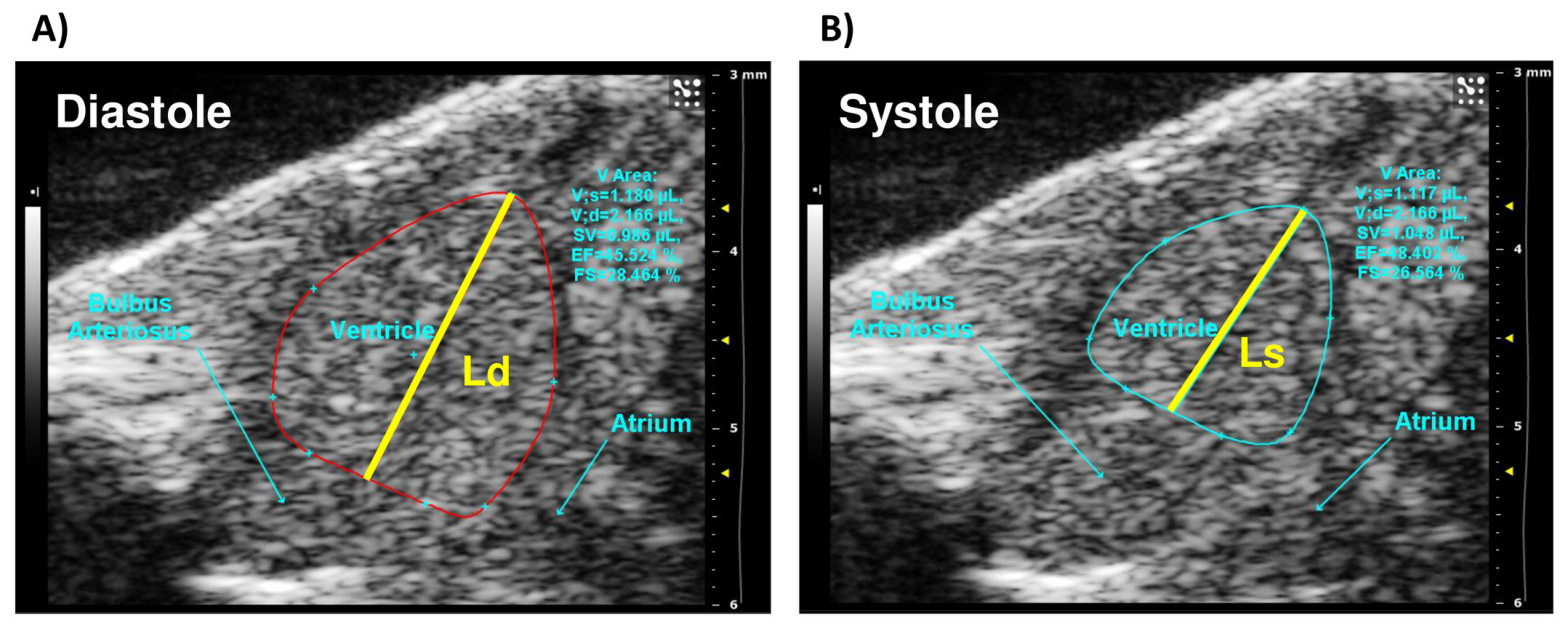
B-mode image of a long-axis view of a WA ZF heart at 28°C. The perimeter of the ventricle represented by (A) red trace for diastole and (B) cyan trace for systole. V Area, ventricular area; V, volume; Ld, greatest length of the spline during diastole; Ls, greatest length of the splice during systole; SV, stroke volume determined by diastolic volume-systolic volume; EF, ejection fraction determined by SV/diastolic volume; FS, fractional shortening determined by (Ld—Ls) / Ld * 100.

**Fig 3:**
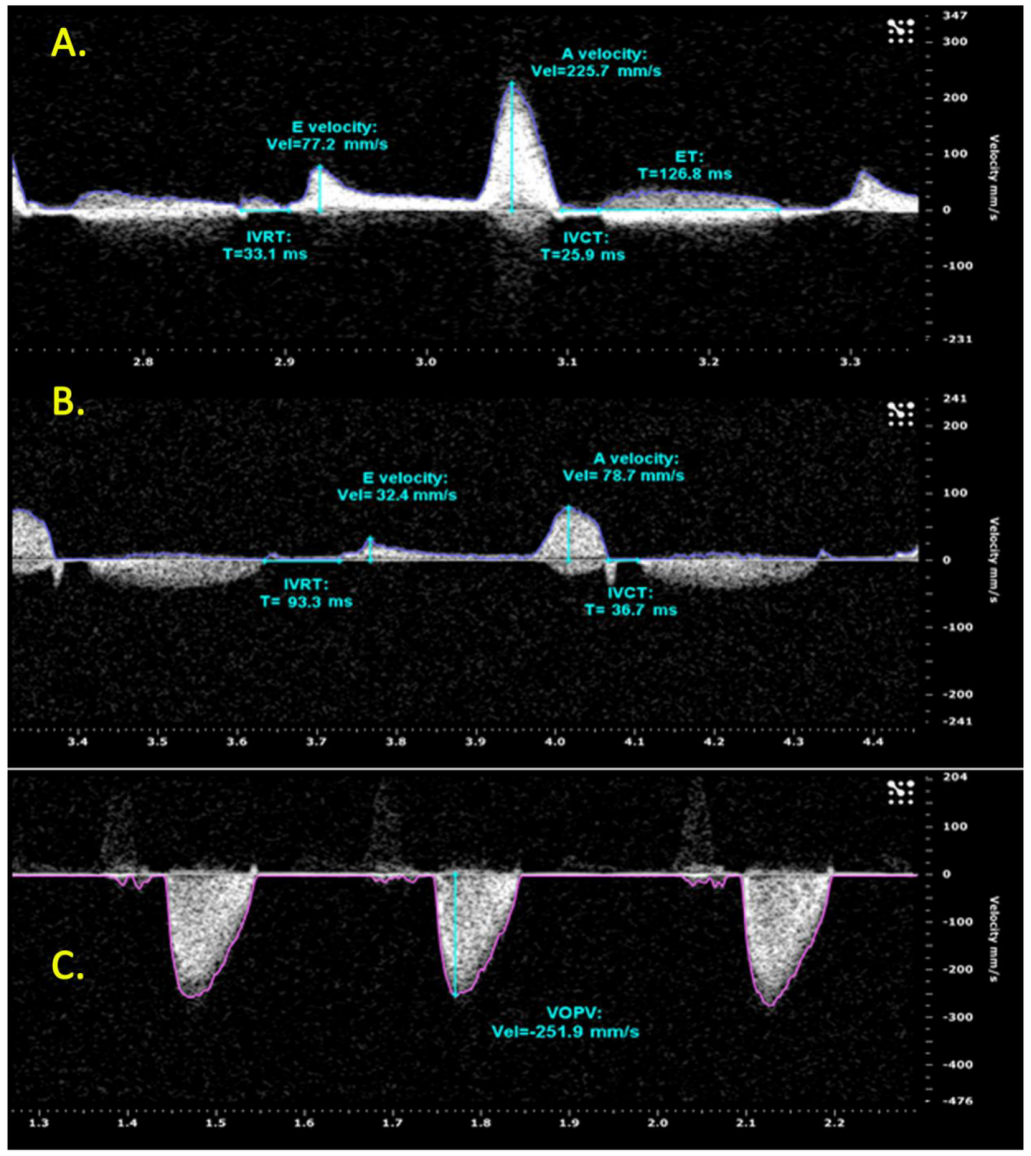
Pulse wave Doppler image of ventricular inflow and outflow velocity. Ventricular inflow image was used to measure IVRT, IVCT, ET, early (e) filling peak velocity and atrial (a) filling peak velocity in a WA ZF at: (A) 28**°**C and (B) 18**°**C. Note the difference in x-axis in the 28**°**C condition and 18**°**C condition. (C) Ventricular outflow peak velocity (VOPV) of a WA ZF at 28**°**C. The Y-axis indicates velocity (mm/s) and the x-axis indicates time (ms). ET, ejection time; IVCT, isovolumetric contraction time; IVRT, Isovolumetric relaxation time; Vel, velocity.

Cardiac functional parameters such as SV, ejection fraction (EF), fractional shorting (FS) and cardiac output (CO) were obtained from a clear two chamber B-mode image (S1–S9 Videos). Ventricular volume was determined from the differences between systolic and diastolic ventricular areas. This was measured by the rotational volume of the ventricular trace around the long axis line of the spline, referring to the long axis line length that extends from the base to the farthest extent of the spline through systole (L_s_) and diastole (L_d_) (Fig 2). Fractional shortening (FS) was calculated by (Ld—Ls) / Ld multiplied by 100 and is illustrated in Fig 2. It should be noted that in clinical echocardiography FS is conventionally measured from the parasternal short axis M-mode image for highest accuracy. However, because of the small size of the ZF heart (~1.5 mm), clear and reliable M mode images were not readily obtainable. Thus we used the parasternal long axis B-mode image and the FS calculations in this study are based upon the diameter from apex to base in diastole and systole and, therefore, represent more the longitudinal contribution to FS. Stroke volume (SV) was measured by the difference between diastolic volume and systolic volume, while ejection fraction (EF) was measured by SV divided by diastolic volume multiplied by 100. The color Doppler and pulsed wave Doppler images were also captured from the same longitudinal plane (S10–S18 Videos). Heart rate, ventricular inflow and ventricular outflow peak velocity were calculated from the pulse wave Doppler images (Fig 3). These measurements were acquired at the point between the atrium and the ventricle for inflow velocity, and between the ventricle and the bulbous arteriosus for outflow velocity. The direction of blood flow was determined using color Doppler, meaning the angle used for quantification of peak velocity can be measured precisely in each fish. The Doppler velocity of the ventricular inflow includes two components: early (E) filling peak velocity that occurs during ventricular relaxation and the atrial (A) filling peak velocity that is the consequence of atrial contraction. Isovolumetric relaxation time (IVRT), isovolumetric contraction time (IVCT) and ejection time (ET) (measured in ms) were also collected from pulsed wave Doppler images of ventricular inflow (Fig 3). IVRT was taken pre-early (E) filling peak and IVCT was taken post-atrial (A) filling peak. MPI was calculated from (IVCT+IVRT) / ET.

Every parameter was determined as the average measurement of at least five cardiac cycles. Each acclimated fish was first measured at their respective acclimated temperature (i.e. 18°C for CA and 28°C for WA). After the first image was acquired, the temperature of the chamber was gradually changed over the course of twelve minutes from 28 to 18°C for WA, and then back to 28°C over the same time span. The same procedure was followed in reverse for CA. The ultrasonic images were taken in a stepwise manner at temperature points of 18 and 28°C. The temperature was maintained for 2 minutes before echocardiographic images were acquired to ensure adequate cooling or warming of the zebrafish body. For three of the 20 zebrafish used in this study, an additional 7.5 ppm of both MS-222 and isoflurane were added in order to maintain the anesthetic condition while the temperature was raised to 28°C without any apparent cardiodepressant effects.

### Statistics

Statistical significance in acute temperature responses for echo parameters were determined using a one-factor two way ANOVA with a randomized block design. The acclimation state was treated as a blocking variable allowing the potential interaction of this effect with temperature to be explored. If there was no interaction, the statistical analysis was redone without the interaction term. If the interaction was significant, acclimation state has a confounding effect which cannot be dissociated from the effect temperature change on the response variable. Values outside of two standard deviations from the mean were discarded from the analysis. A *p*-value of less than 0.05 was accepted as statistically significant. The JMP 11 software package was used for all statistical analysis.

## Results

### Functional analyses

**Fig 4:**
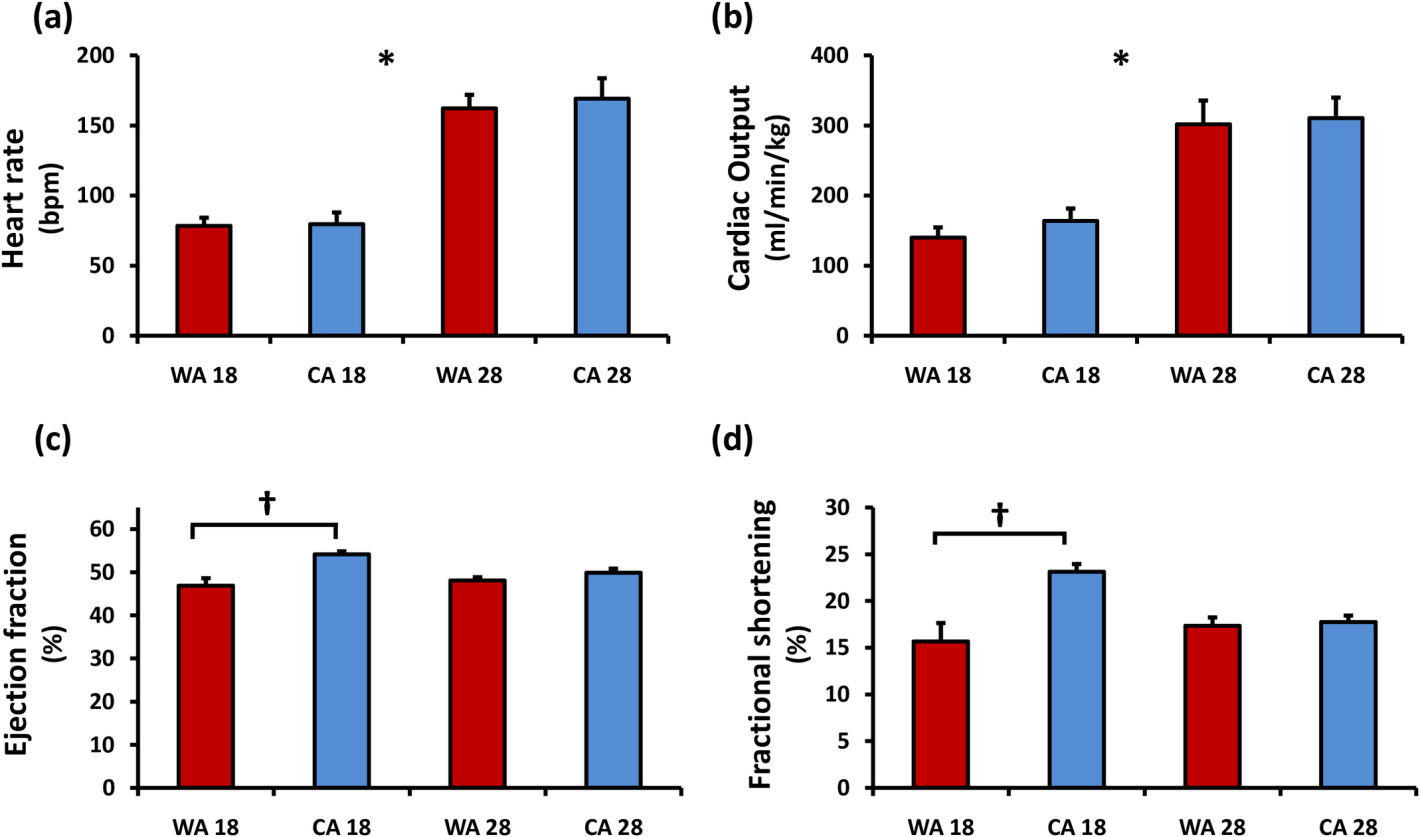
Ventricular functional parameters were calculated from B-mode long-axis plane (n = 10). (A) Heart rate, (B) normalized cardiac output, (C) ejection fraction and (D) fractional shortening of both WA and CA zebrafish at two acute temperatures. Error bars represent SEM. * represents significant difference of both groups between temperatures. † represents significant interacting effect of acclimation state. Red: warm acclimated (WA) zebrafish. Blue: cold acclimated (CA) zebrafish.

Heart rate was used as the principle parameter to assess whether the anesthetic protocol caused significant cardiac depression that would confound the accurate determination of cardiac function. The combination of MS-222 and isoflurane kept the heart rate above 160 bpm in fish at 28°C. An acute temperature decrease of 10°C significantly decreased heart rate by approximately half (*p*<0.0001, Fig 4A). Cold acclimation did not significantly modify the acute temperature heart rate response, as there was no significant difference in heart rates taken at both 28°C (162–169 bpm) and 18°C (78–79 bpm) for both CA and WA fish. SV did not fluctuate significantly with acute temperature perturbation and cold acclimation had no statistically significant effect on this response (1.06–1.17 μl). Combining these two factors, CO was significantly increased by about two-fold for both groups in increasing the acute temperature from 18 to 28°C (*p*<0.0001) and acclimation state had no significant effect on the magnitude of CO at either temperature (Fig 4B).

Ejection fraction, the volumetric fraction of blood pumped out of the ventricle with each cardiac cycle relative to the maximum volume in the heart after diastole (EDV), can be used as an index of contractility (Table 1). Acute temperature perturbation did not modify EF significantly in both groups, but acclimation state had a confounding effect on the temperature response (cross-effect *p* = 0.018). With CA, EF was higher at both temperatures (18 and 28°C) and increased when temperature was decreased to 18°C (Fig 4C).

**Table 1:** Parameters from echocardiographic B-mode of the zebrafish heart for WA vs CA fish at 18°C and 28°C.

Group	Temp(°C)	HR(bpm)		BW(g)		SV(μl)		EF(%)		FS(%)		ESV(μl)		EDV(μl)	
		mean	SEM	mean	SEM	mean	SEM	mean	SEM	mean	SEM	mean	SEM	mean	SEM
**WA**	**18**	**78**	6	**0.6**	0.03	**1.1**	0.1	**46.9**	1.7	**15.7**	2.0	**1.2**	0.1	**2.3**	0.2
	**28**	**162**	10			**1.1**	0.1	**48.1**	0.8	**17.3**	0.9	**1.2**	0.1	**2.3**	0.2
**CA**	**18**	**80**	8	**0.6**	0.10	**1.2**	0.1	**54.2**	0.7	**23.1**	0.8	**1.0**	0.1	**2.2**	0.3
	**28**	**169**	15			**1.1**	0.1	**49.9**	0.9	**17.7**	0.7	**1.0**	0.1	**2.1**	0.3
		*		**0.58**				**†**		**†**					

HR, heart rate; BW, body weight; SV, stroke volume; EF, ejection fraction; FS, fractional shortening; ESV, end systolic volume; EDV, end diastolic volume.

* represents significant difference between temperatures within group *p*<0.05.

† represents significant interacting effect of the acclimation state *p*<0.05.

Fractional shortening (FS), the degree of shortening of the ventricle between end-diastole and end-systole, is another index of myocardial contractility. Acute temperature change did not change FS significantly, but the acclimation state did have a confounding effect on the temperature response (cross-effect p = 0.006). At 28°C, both CA and WA had similar FS (≈17%), but at 18°C CA fish had a significant higher FS (CA = 23% vs WA = 16%—Fig 4D).

In the WA group neither end systolic volume (ESV) nor EDV changed significantly with acute temperature fluctuation (ESV of 1.24 μl and 1.19 μl, EDV of 2.34 μl and 2.31 μl at 18°C and 28°C, respectively). With CA, both ESV and EDV show a lower trend (ESV of 0.99 μl and 1.05 μl, EDV of 2.16 μl and 2.11 μl at 18°C and 28°C, respectively) but there was no significant effect of acclimation state on the acute temperature fluctuation response seen in either parameter (Table 1). It is important to note that SV in some studies is normalized to body weight and denoted as μl/g. This is a common normalization procedure seen in many teleost studies (goldfish [22]; flounder [23]) due to the correlation between blood volume and body weight. However, since no significant changes in body weight were seen between cold-acclimated and warm-acclimated fish used in the echocardiography study the data are shown in absolute values. For comparison with other studies and species, normalized CO is displayed as ml min^-1^ kg^-1^ in Fig 4.

### Pulse-wave Doppler measurements

**Fig 5:**
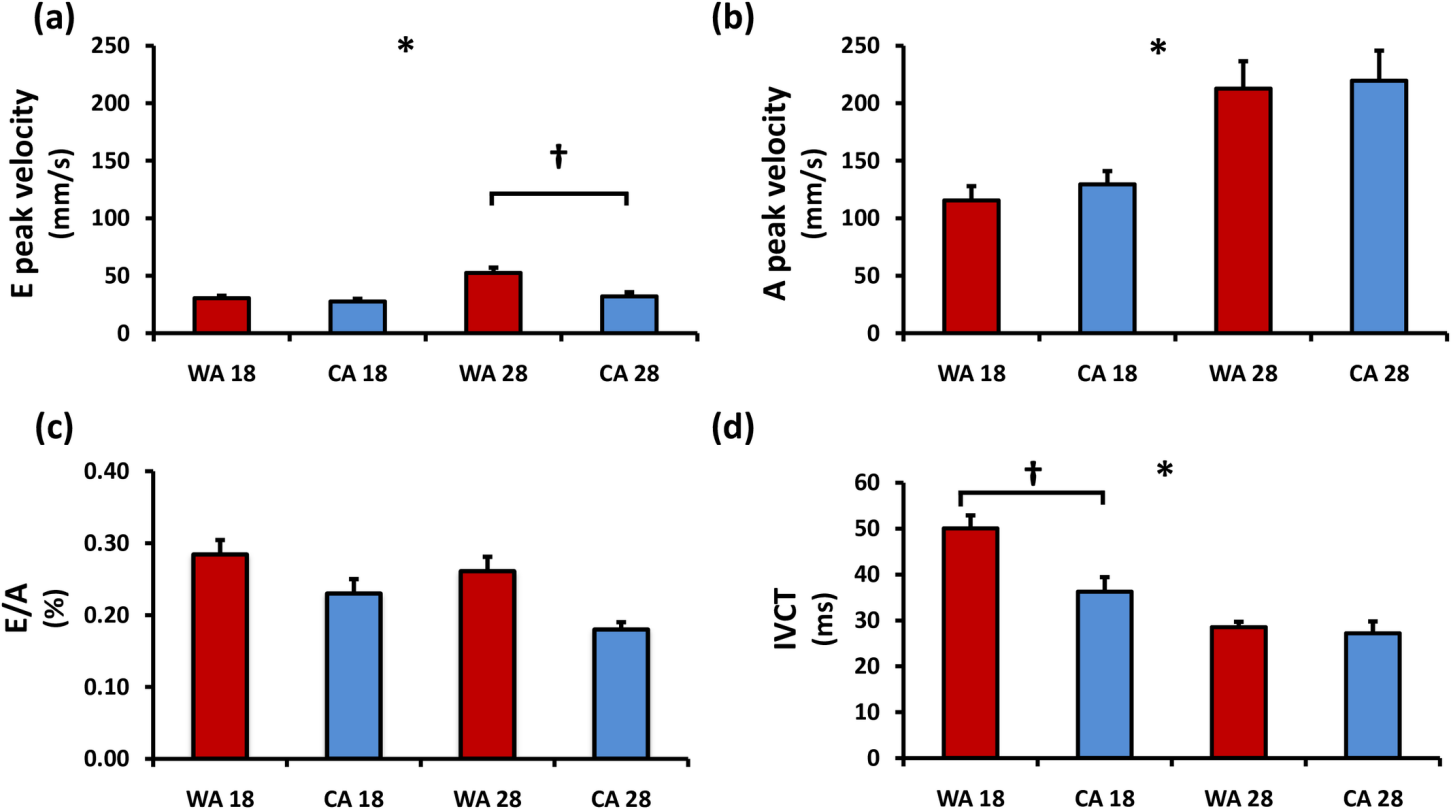
Parameters were calculated from pulse wave Doppler mode of ventricular inflow view (n = 10). (A) Early (e) filling peak velocity, (B) atrial (a) flow peak velocity, (C) E/A ratio and (D) isovolumic contraction time (IVCT) of CA and WA zebrafish at two acute temperatures. The ratio of early (e) filling peak velocity and atrial (a) late filling peak velocity allows for evaluation of zebrafish ventricular function. Error bars represent SEM. * represents significant difference of both groups between temperatures. † represents significant interacting effect of acclimation state. Red: warm acclimated (WA) zebrafish. Blue: cold acclimated (CA) zebrafish.

Ventricular inflow velocities including E and A (early and atrial) peak velocity and ventricular outflow velocities were significantly greater at 28°C (*p*<0.0001—Table 2) in both groups. There were no significant differences in A peak or V outflow velocities between WA and CA fish when measured at either 18°C or 28°C. There was a confounding effect of acclimation state on E peak velocity temperature response, shown as WA fish at 28°C having a greater E peak velocity than CA fish (WA 52.34 mm/s vs CA 32.02 mm/s, p = 0.004, Fig 5A). Acute temperature decrease significantly lengthens the duration of both the E phase (*p* = 0.0076) and A phase (*p*<0.0001). The change in duration of only the A phase is confounded by acclimation state (Table 2A). Both E duration and A duration are part of the overall increase in inflow time with acute temperature decrease (*p* = 0.0007). When normalized to beat duration (HR^-1^), the acute temperature no longer significantly affected duration time (Table 2B).

**Table 2:** Parameters from pulse wave Doppler and colour Doppler imaging of ZF heart.

A.													
	**Temp(°C)**	**E peak**		**A peak**		**Outflow**		**E flow**		**A flow**		**Inflow**	
**Group**		**Velocity (mm/s)**						**Duration (ms)**					
		**mean**	SEM	**mean**	SEM	**mean**	SEM	**mean**	SEM	**mean**	SEM	**mean**	SEM
**WA**	**18**	**30.5**	2.1	**115.6**	12.3	**126.5**	17.1	**290.3**	68.2	**118.2**	3.3	**408.5**	68.7
	**28**	**52.4**	4.7	**212.7**	23.9	**256.4**	32.9	**139.7**	16.3	**58.9**	1.0	**198.7**	16.6
**CA**	**18**	**27.7**	2.3	**129.5**	11.5	**128.7**	11.2	**354.1**	87.6	**102.8**	1.4	**456.9**	86.6
	**28**	**32.0**	3.6	**219.6**	26.3	**193.5**	10.9	**162.9**	44.9	**57.3**	2.1	**220.2**	44.9
		*†		*		*		*		*†		*	
**B.**													
						**Duration Normalized by Beat Duration**							
**Group**	**Temp(**^**o**^**C)**	**Beat duration(ms)**				**E flow(ms)**		**A flow(ms)**			**Inflow(ms)**		
		**mean**		SEM		**mean**	SEM	**mean**		SEM	**mean**		SEM
**WA**	**18**	**814.0**		74.3		**32.1**	4.5	**15.4**		1.2	**47.5**		3.5
	**28**	**381.7**		22.0		**35.7**	2.4	**15.9**		0.8	**51.5**		1.9
**CA**	**18**	**834.2**		88.5		**36.9**	6.7	**13.7**		1.5	**50.6**		5.3
	**28**	**387.2**		42.6		**36.5**	6.0	**16.1**		1.3	**52.6**		4.7
		*											
**C.**													
**Group**	**Temp(**^**o**^**C)**	**IVCT(ms)**			**IVRT(ms)**			**ET(ms)**			**MPI(%)**		
		**mean**		SEM		**mean**	SEM	**mean**		SEM	**mean**		SEM
**WA**	**18**	**50.1**		2.8		**110.4**	4.2	**236.0**		9.3	**0.69**		0.04
	**28**	**28.5**		1.2		**39.3**	1.8	**116.5**		7.0	**0.60**		0.04
**CA**	**18**	**36.2**		3.2		**116.7**	4.3	**246.7**		11.7	**0.62**		0.05
	**28**	**27.2**		2.6		**42.5**	1.6	**111.2**		5.1	**0.64**		0.04
		*†				*		*					

**A.** Ventricular inflow, made up of two components: early (E) filling peak velocity occurring during ventricle relaxation (E peak vel in mm/s) and the atrial (A) filling peak velocity resulting from active atrial contraction (A peak vel in mm/s), and ventricular outflow peak velocity (V outflow vel in mm/s). Early flow duration (E duration in ms), late flow duration (A duration in ms) make up the total inflow duration. **B.** Normalized duration times. Duration is expressed as proportion of beat duration (ms) calculation as the inverse of HR. **C.** Isovolumic contraction time measuring systolic performance (IVCT) in ms, isovolumic relaxation time measuring diastolic performance (IVRT) in ms and ejection time (ET) in ms are also measured from this view. The myocardial performance index (MPI) is a Doppler-derived time interval index that combines both systolic and diastolic cardiac performance (IVCT + IVRT)/ET.

* represents significant difference between temperatures p<0.05.

† represents significant interacting effect of acclimation state p<0.05.

### E/A values

No significant variation in the E/A ratio was found with acute temperature fluctuation. E/A ratios were comparable at either temperature in WA fish (E/A ≈ 0.28). While the effects of acclimation were not confounding with those of acute temperature response, a trend of lower E/A ratios appeared in CA fish (Fig 5C).

### The myocardial performance index (MPI)

The myocardial performance index (MPI) is a pulse wave Doppler-derived time interval index that incorporates both systolic and diastolic time intervals and is impacted by both systolic and diastolic cardiac function. Both systolic and diastolic dysfunction can result in abnormality in myocardial relaxation which can prolong the relaxation period (isovolumic relaxation time, IVRT). Systolic performance, reflected in part by the isovolumic contraction time (IVCT) was longer at 18 vs. 28°C for both WA and CA fish (*p*<0.0001). Acclimation state did affect the acute temperature response (cross-effect *p* = 0.019). IVCT was longer in WA fish relative to CA at both 18°C and 28°C (Fig 5D). Diastolic performance, reflected in part by the isovolumic relaxation time was also longer at 18 vs. 28°C, a significant effect from the acclimation condition (*p*<0.0001). However, while IVRT appeared to be longer in CA fish, this trend was not statistically significant. Ejection time (ET) was also significantly decreased with an acute temperature decrease to 18°C (*p*<0.0001) but was unaffected by acclimation state. Overall, the MPI did not vary at either acute temperature condition, and there was no acclimation effect (Table 2C).

## Discussion

In this study high frequency echocardiographic measurements allowed us to obtain a critical assessment of *in vivo* cardiac function in zebrafish as a function of temperature. The zebrafish has been used in the literature as a robust model of vertebrate cardiac development and function because of the availability of annotated genomic information, accessibility of numerous mutant strains and the transparency of the developing embryos that permits measurement of numerous cardiac parameters by visual inspection. Due to a loss of transparency in adulthood as well as the small size of adult zebrafish hearts (1–2 mm in diameter), it is challenging to measure all facets of cardiac function *in vivo* without sophisticated imaging techniques. Detailed, reproducible measurements using high-frequency echocardiography in zebrafish can be taken only with the use of a very high frequency probe (70 MHz) and extreme diligence. In the literature, variability exists in *in vivo* fish cardiac function results, likely due to the method of anesthetization and restraint of fish in addition to lower resolution echocardiographic measurements. The anesthetic protocol used in this study, modified from Huang et al. [21], allows for prolonged state of anesthesia with a significantly lower impact on both heart rate (HR) and ejection fraction (EF) [21]. All fish maintained a minimal EF of 40% throughout the experiment. When the acute temperature changes were expressed as Q_10_:

$$\text{where}\;Q10=\frac{R2}{R1}{\text{exp}}^{\frac{10}{T2-T1}}$$

or simply the fold-change in activity per 10°C) the data fell in the range of 2.2. This is well within the expected normalized rate change of two-to three-fold per 10°C change for most physiological processes. The echocardiographic measurements in this study show a similar range in HR *in vivo*, as well as a comparable change in HR with cooling [24]. Overall, these inclusionary criteria suggest cardiac function was maintained to ideal conditions to monitor changes in response to a stressor such as temperature change.

### Heart rate is the primary cardiac function response to acute temperature changes

Resting HR in ectotherms is greatly influenced by temperature [25,26]. Typically, teleosts cope with acute temperature changes by modifying HR to maintain CO (zebrafish [27]; cod [2]; salmon [5]; flounder [28]). Most temperate fish exhibit resting heart rate increases with increased temperatures on the average with a Q_10_ of ~2.0 [29]. In this study, an acute decrease of temperature from 28°C to 18°C resulted in a 48% decrease in HR, demonstrating a Q_10_ of 2.2. Stroke volume, the other determinant of CO, undergoes very little change in response to acute temperature perturbations with a Q_10_ of ~1.0 [1,2,5,28]. Modulations in HR alone appear to be responsible for the changes in cardiac output reported in this study (Q_10_ = 2.2). These results corroborate what has been suggested from studies with other teleost species [27] using *in situ* and other approaches. EDV was unchanged by cooling from 28°C to 18°C, similar to what has been reported with variable temperatures in measurements from other teleosts [10,30] using other methodologies. Increased EDV would have a particularly important role in determining CO in fish, as it may reduce the scope for increasing SV [10,30,31,32]. The lack in change of both SV and EDV with cooling supports the notion that the cardiac output of zebrafish is controlled, to a great extent, by HR in response to acute temperature fluctuations.

Ventricular inflow and outflow velocities were both significantly increased at 28°C relative to 18°C (Q_10_−1.8 and 2.0, respectively; Table 2). Our data showed a slightly higher ventricular outflow than ventricular inflow at 28°C (213 mm sec^-1^ inflow vs 256 mm sec^-1^ outflow) and similar velocities for both inflow and outflow at 18°C (115.6 mm/sec inflow and 126.5 mm sec^-1^ outflow). Sun et al [19] found peak ventricular outflow (800 mm sec^-1^) to be significantly less than peak ventricular inflow (1440 mm sec^-1^), demonstrating the opposite of what was seen in this study. In both the Sun et al. and Hu et al. [19,33] studies, velocity measurements were determined at a lower temperature range (15–25°C) with high to very high doses (100 to >200 ppm) of MS-222 as an anesthetic. Any alteration in flow duration with acute temperature change corresponded with fluctuations in beat duration, or inversely proportional to HR (Table 2B), both of would be affected by MS-222. As well, our study optimized the locations to measure velocity (the point between atrium and ventricle for inflow velocity and between ventricle and bulbus for outflow velocity) and used color Doppler for visualization of the direction of blood flow to improve the resolution of measurement. This technique of combining color Doppler with pulse-wave Doppler is more user-friendly than using only pulse-waved Doppler where estimation of an average angle is required for each zebrafish [34,35]. The results presented here demonstrate Q_10_ values for chamber-to-chamber velocities are internally consistent with variations in other parameters measured (HR, CO).

### Zebrafish CO shows no response to cold acclimation

In the current study acclimation had no confounding effect on CO, either through HR or SV. Both CA and WA fish had comparable HR at 18°C and 28C, suggesting that the HR response to the current ambient temperature outweighs any acclimated response. SV is highest when the ambient temperature matches the acclimation temperature in this study, such as SV in the CA group is higher at 18°C while SV in the WA group decreases upon cooling to 18°C (Table 1). The variation between the two resulted in a slightly greater Q_10_ for CO in WA fish compared to CA fish (2.15 vs 1.89). This suggests that there is a slightly greater compensation over the 10°C acute change is made by WA fish. While SV may be altered with cold acclimation of sufficient duration and temperature change in many teleost species [8,36,37,38], this does not appear to be an acclimation response. These results correspond to overall lack of ventricular hypertrophy in zebrafish measured histologically in other studies [39], in contrast with larger hearts of cold-acclimated trout exhibiting enhanced SV [40,41]. The possibility of species-specific responses to acclimation makes characterization of functional properties of the heart in adult zebrafish crucial as its role as a model for cardiac function expands.

While changes in HR with acute temperature change are reflected in both IVCT and IVRT, these measures did display a confounding effect of acclimation state (Table 2). Relaxation time calculated as IVRT is longer with an acute temperature decrease and tends to be lengthened in cold-acclimated fish though not to a significant extent. Predictably, in acute temperature fluctuation conditions, contraction time calculated as IVCT is longer at 18°C. This acute temperature response is confounded by acclimation state, with CA fish exhibiting shorter IVCT at both temperatures. The biggest difference is that CA fish demonstrated a much shorter IVCT than WA fish at warm temperatures. This could be indicative of a greater degree of difficulty for ectotherms to deal with temperature increase compared to temperature decrease [42,43,44].

In cardiac muscle, cold-acclimation can extensively modify expressions of genes involved with protein synthesis and hypertrophy as well as genes encoding for contractile proteins [39,45,46,47,48]. In zebrafish specifically, the acclimation protocol used in this study increases the mRNA levels of cardiac troponin C (cTnC) with decreased temperature, a critical component of the cardiac contractile element [46]. Conditional antisense knockdown of cTnC in zebrafish results in a significantly lower EF [49], a common index of contractility [24]. This study revealed a significant increase in EF with cold acclimation, which is consistent with an increase in the expression of contractile proteins such as cTnC [46]. Importantly, changes to EF were only observed at cold temperatures, where CA fish at 18°C had significantly higher EF than CA fish at 28°C or WA fish at either temperature. This suggests that tissue remodeling optimized the contractility in CA fish to function at 18°C even if overt morphological changes were not seen. Using histological techniques, other zebrafish studies have shown no change in heart volume but a decrease in the compact myocardium with cold acclimation [39]. Increases in compact myocardium is suspected to be compensation for decreased power-generating ability or simply increased activity levels at higher temperatures [45], and may also account for a lack in change of size of the ventricle as a whole. Further work will need to be done to link potential changes in contractility with the molecular mechanisms of remodelling specific to zebrafish.

### Filling patterns do not change in response to acute temperature or acclimation

In this study, the E/A ratio was found to be 0.28 regardless of the temperature. This value corroborates previous zebrafish studies, which have shown similar E/A ratios at 15°C [12,19], 22°C [50] and 25°C. The E/A ratio accounts for the two components to the preload of the ventricle, early filling created by E-flow and late filling created by A-flow which is atrial-contraction dependent (Fig 5). These E/A ratios indicate that late diastolic filling caused by atrial contraction in zebrafish account for the majority of ventricular filling regardless of the temperature. In mammals, this phase is created by venous pressure and under resting conditions is the main determinant of ventricular filling. Ventricular inflow in fish, unlike that in mammals, is primarily due to atrial contraction (A phase) [51], seen as a larger A-phase peak velocity compared to the E-flow. This model of filling is supported by the similarity between atrial and ventricular volume [19,51,52], and the favorable filling pressure gradient that exists only during atrial systole [53]. However, one of the consequences of the low E velocities in the ZF heart is a greater difficulty in its accurate assessment which may contribute to a lower power for comparison. Despite this it was clear that both E-flow and A-flow significantly decrease as temperature changes from 28°C to 18°C with comparable Q_10_ values (A peak velocity 1.84; E peak velocity 1.71). Acute reduction in temperature has been shown to have larger effects in atrial versus ventricular action potential duration [24], but this does not appear to cause a difference in the proportion of active atrial contraction that contributes to EDV. Since both E and A flow change with temperature, the E/A ratio is an important parameter in evaluating cardiac function for comparison across species and between studies.

Acclimation state did have statistical effect on E/A fraction with decreased ratios seen with cold acclimation. This E/A ratio suggests that cold-acclimated fish rely more on active atrial contraction to maintain SV. There was a significant cross-effect of acclimation on E-peak velocity response to acute temperature. CA fish had lower E-flow. The Q_10_ for the change in E-flow velocity in WA fish was 1.71, but in CA fish was only 1.15. Lack of compensation in passive filling patterns with acclimation is suggested by this ratio and could also be reflective of a less compliant ventricle suggested by EDV and IVRT in CA. Compliance of the zebrafish ventricle has been suggested to be lower than that of human hearts [34] this is in turn influenced by temperature. Our current results support the notion that the passive properties of the ventricle may be influenced by acclimation even with no change in ventricular size, such as is seen in remodelling of myocardial layers seen with acclimation in other zebrafish studies [39]. An increase in temperature is more stressful for an ectothermic fish to maintain cardiac output [42] which may be responsible for CA fish facing acute temperature increase to slightly modulate E/A ratio. In fact, CA fish at 28°C demonstrate the lowest E/A ratio, possibly indicating ventricular dysfunction. With a larger EF in CA as well as a slightly larger EDV, there is a possibility of a greater atrial contraction or simply a longer cardiac cycle suggested by IVRT.

## Summary

This study allowed for the critical assessment of cardiac parameters in the intact zebrafish in response to both acute and long-term temperature change using high-resolution echocardiography. Modulations in HR dictate changes in cardiac output in response to acute temperature changes in both WA and CA fish. SV was highest when the ambient temperature matches the acclimation temperature. All other functional parameters were maintained throughout the acute temperature challenge. Cold acclimation improved systolic function at 18°C in comparison to the WA group with increases in both EF and FS and decreases in IVCT. The decreased E velocity and E/A ratio in the CA group may be associated with increased reliance on atrial contraction. However, the consistency of the E/A ratio with respect to temperature shows it is an important parameter in evaluating cardiac function for comparison between studies in zebrafish. The accuracy of the *in-vivo* high-resolution echocardiography used in this study lends confidence to our understanding of responses of teleost cardiac function to environmental perturbation. Species-specific responses to both acute temperature changes and longer-term acclimation make this high-resolution characterization of functional properties of the heart critical for the expansion of the adult zebrafish as a model for cardiac function.

## Supporting Information

S1 Table The comparison between present and other studies. (DOCX)

S1 Video Longitudinal axis B-mode video of a warm-acclimated (WA) zebrafish at 28oC. Atrium, ventricle and bulbus arteriosus are visible in this plane.
(M4V)

S2 Video Longitudinal axis B-mode video of a warm-acclimated (WA) zebrafish at 28oC. (M4V)

S3 Video Longitudinal axis B-mode video of a warm-acclimated (WA) zebrafish at 28oC. (M4V)

S4 Video Longitudinal axis B-mode video of a warm-acclimated (WA) zebrafish at 18oC. (M4V)

S5 Video Longitudinal axis B-mode video of a warm-acclimated (WA) zebrafish at 18oC. (M4V)

S6 Video Longitudinal axis B-mode video of a cold-acclimated (CA) zebrafish at 28oC. (M4V)

S7 Video Longitudinal axis B-mode video of a cold-acclimated (CA) zebrafish at 28oC. (M4V)

S8 Video Longitudinal axis B-mode video of a cold-acclimated (CA) zebrafish at 18oC. (M4V)

S9 Video Longitudinal axis B-mode video of a cold-acclimated (CA) zebrafish at 18oC. (M4V)

S10 Video Longitudinal axis color-Doppler mode video of a warm-acclimated (WA) zebrafish at 28oC. Color indicates the direction of blood flow. Red flow represents the ventricular inflow toward the transducer while blue flow represents the ventricular outflow away from the transducer.
(M4V)

S11 Video Longitudinal axis color-Doppler mode video of a warm-acclimated (WA) zebrafish at 28oC. (M4V)

S12 Video Longitudinal axis color-Doppler mode video of a warm-acclimated (WA) zebrafish at 28oC. (M4V)

S13 Video Longitudinal axis color-Doppler mode video of a warm-acclimated (WA) zebrafish at 18oC. (M4V)

S14 Video Longitudinal axis color-Doppler mode video of a warm-acclimated (WA) zebrafish at 18oC. (M4V)

S15 Video Longitudinal axis color-Doppler mode video of a cold-acclimated (CA) zebrafish at 28oC. (M4V)

S16 Video Longitudinal axis color-Doppler mode video of a cold-acclimated (CA) zebrafish at 28oC. (M4V)

S17 Video Longitudinal axis color-Doppler mode video of a cold-acclimated (CA) zebrafish at 18oC. (M4V)

S18 Video Longitudinal axis color-Doppler mode video of a cold-acclimated (CA) zebrafish at 18oC. (M4V)
