# Correction: Functional Assessment of Cardiac Responses of Adult Zebrafish (*Danio rerio*) to Acute and Chronic Temperature Change Using High-Resolution Echocardiography

**DOI:** 10.1371/journal.pone.0149741

**Published:** 2016-02-12

**Authors:** Ling Lee, Christine E. Genge, Michelle Cua, Xiaoye Sheng, Kaveh Rayani, Mirza F. Beg, Marinko V. Sarunic, Glen F. Tibbits

S3 Video is a duplicate of S5 Video. Please view the correct S3 Video below.

## Supporting Information

S3 Video Longitudinal axis B-mode video of a warm-acclimated (WA) zebrafish at 28oC. (MP4)
