# Supplementary material for: Functional Assessment of Cardiac Responses of Adult Zebrafish (Danio rerio) to Acute and Chronic Temperature Change Using High-Resolution Echocardiography
Source: PLoS One. 2016 Jan 5;11(1):e0145163. doi: 10.1371/journal.pone.0145163 (PMC4701665; doi:10.1371/journal.pone.0145163)
Supplement: S1 Table — (DOCX) [file pone.0145163.s001.docx]

**S1 Table. The comparison between present and other studies.**

| Reference | | | Present study | | | Lee et al. (Ref 34) | | | Kang et al. (Ref 29) | Hein et al. (Ref 20) | | |
| --- | --- | --- | --- | --- | --- | --- | --- | --- | --- | --- | --- | --- |
| **Experimental design and instrumentation** | | | | | | | | | | | | |
| Experimental design | | | Echo imaging for warm-acclimated (WA) and cold acclimated (CA) at 18 and 28 °C. | | | Cryo-cauterization to the ZF ventricular epicardium | | | ZF Doppler imaging was performed prior to and after ventricular amputation. | Cryo-cauterization to the ZF ventricle. Cardiac performance was serially evaluated for up to 120 days. | | |
| Instrumentation | | | VisualSonics Vevo 2100  MS-700 - linear array ultrasound transducer | | | Custom US array imaging system with 256-element linear array transducer. | | | Custom 64-channel US imaging with 256-element linear array transducer | VisualSonics Vevo2100  MS-550D – linear array ultrasound transducer | | |
| Ultrasound frequency | | | 30-70 MHz | | | 30 MHz | | | 30 MHz | 22-55 MHz | | |
| **Experimental animals and experimental conditions** | | | | | | | | | | | | |
| ZF body length (cm) | | | 3.85 ± 0.27 | | | 3.0–4.0 | | | 4.08 ± 0.36 | 2.5–4.0^a^ | | |
| ZF Genotype | | | WT | | | WT | | | WT | WT^b^ | | |
| N | | | 10 | | | 6 | | | 5 | 11 | | |
| Temperature | | | 18 and 28 °C | | | 28°C | | | 26°C | 22^o^C^c^ | | |
| Anesthetic  (ppm) | | | Initial: 30 Tricaine  + 30 isoflurane  Maintain: 45 Tricaine  + 45 isoflurane | | | Initial: 400 Tricaine  Maintain: 200 Tricaine | | | Initial: 800 Tricaine  Maintain: 400 Tricaine | 0.44 Phenoxyethanol^d^ | | |
| HR (bpm)  Q_10_= 2.15 | | | 18°C: 78  28°C: 168 | | | 28°C: 140 | | | 26°C: 40^e^  28^o^C: 47^f^ | 22^o^C: 91  28^o^C: 146^f^ | | |
| **B Mode Imaging – long axis plane** | | | | | | | | | | | | |
| ESV  (µl) | | 18°C: 1.24  28°C: 1.19 | | | ND | | | ND | | | ND | |
| EDV  (µl) | | 18°C: 2.34  28°C: 2.31 | | | ND | | | ND | | | ND | |
| Systolic area (mm^2^) | | ND | | | ND | | | ND | | | 22^o^C: 0.34 | |
| Diastolic area (mm^2^) | | ND | | | ND | | | ND | | | 22^o^C: 0.60 | |
| SV (µl) | | 18°C: 1.10  28°C: 1.12 | | |  | | | ND | | | 22^o^C: 0.23 | |
| EF (%) | | 18°C: 47  28°C: 48 | | | ND | | | ND | | | 22^o^C: 54 | |
| FS (%) | | 18°C: 16  28°C: 17 | | | ND | | | ND | | | 22^o^C: 24 | |
| CO (µl/min) | | 18°C: 59.50  28°C: 127.60 | | | ND | | | ND | | | 22^o^C: 20.93 | |
| **B Mode Imaging – short axis plane** | | | | | | | | | | | | |
| Systolic area (mm^2^) | | ND | | | ND | | | ND | | | 22^o^C: 0.39 | |
| Diastolic area (mm^2^) | | ND | | | ND | | | ND | | | 22^o^C: 0.69 | |
| FAC (%) | | ND | | | ND | | | ND | | | 22^o^C: 42.98 | |
| **PW/Tissue Doppler mode – long-axis plane** | | | | | | | | | | | | |
| A velocity (mm/s) | 18°C: 116 (Q_10_ = 1.84)  28°C: 213 | | | 28°C: 91 | | | 26°C: 152  28^o^C: 171^g^ | | | | | 22^o^C: 160  28^o^C: 230^g^ |
| E velocity (mm/s) | 18°C: 31 (Q_10_ = 1.70)  28°C: 52 | | | 28°C: 11 | | | 26°C: 20  28^o^C: 22^g^ | | | | | 22^o^C: 32  28^o^C: 44^h^ |
| E/A | 18°C: 0.28  28°C: 0.26 | | | 28°C: 0.12 | | | 26°C: 0.13 | | | | | 22^o^C: 0.21 |
| V outflow velocity (mm/s) | 18°C: 127  28°C: 256 | | | ND | | | ND | | | | | ND |
| A VTI (mm) | ND | | | ND | | | ND | | | | | 22^o^C: 7.10 |
| V VTI (mm) | ND | | | ND | | | ND | | | | | 22^o^C: 9.24 |
| IVCT (ms) | 18°C: 50.1  28°C: 28.5 | | | ND | | | 26°C: 38 (Tissue) | | | | | ND |
| IVRT (ms) | 18°C: 110.4  28°C: 39.3 | | | ND | | | 26°C: 102 (Tissue) | | | | | ND |
| ET (ms) | 18°C: 236.03  28°C: 116.51 | | | ND | | | 26°C: 129.6 (Tissue) | | | | | ND |
| MPI | 18°C: 0.69  28°C: 0.60 | | | ND | | | 26°C: 1.08 (Tissue) | | | | | ND |
| E duration  (ms) | 18°C: 290  28°C: 140 | | | ND | | | ND | | | | | ND |
| A duration  (ms) | 18°C: 118  28°C: 59 | | | ND | | | ND | | | | | ND |
| Inflow time  (ms) | 18°C: 409  28°C: 199 | | | ND | | | ND | | | | | ND |
| V outflow volume (µl) | ND | | | 28°C: 0.028 | | | ND | | | | | ND |
| ΔP across AV (mm Hg) | ND | | | 28°C: 2.5 | | | ND | | | | | ND |
| ΔP across VB (mm Hg) | ND | | | 28°C: 0.7 | | | ND | | | | | ND |

a. body length estimated from age of 3-6 months

b. two genotypes were evaluated but only WT is reported here for comparison purposes

c. temperature was not reported in this paper but was assumed to be room temperature or 22^o^C

d. ppm of 2-Phenoxyethanol was calculated from a concentration of 3.2 µM and a molecular weight of 138.16 g/mol

e. HR was not reported in this paper but was calculated from the PW trace for this comparison

f. HR at 28^o^C calculated from HR at recorded temperature and our determined Q_10_ of 2.15

g. A velocity at 28^o^C calculated from A velocity at recorded temperature and our determined Q_10_ of 1.84

h. E velocity at 28^o^C calculated from E velocity at recorded temperature and our determined of Q_10_ of 1.70
